# Optimizing vedolizumab therapy in ulcerative colitis: A critical synthesis of trial evidence and the emerging role of artificial intelligence

**DOI:** 10.1371/journal.pdig.0001208

**Published:** 2026-02-05

**Authors:** Alfadl Abdulfattah

**Affiliations:** Department of Internal Medicine, College of Medicine, Jazan University, Jazan, Saudi Arabia; National University of Singapore, SINGAPORE

## Abstract

**Background:**

Vedolizumab, a monoclonal antibody targeting the α4β7 integrin, offers gut-selective immunosuppression and represents a cornerstone biologic therapy for moderate-to-severe ulcerative colitis (UC). While pivotal randomized controlled trials (RCTs) have established its efficacy, a substantial subset of patients experience primary non-response. This variability presents significant clinical challenges, including patient morbidity and healthcare costs associated with cycling through ineffective therapies, underscoring an urgent need for personalized treatment strategies.

**Objectives:**

This review aims to critically reappraise the foundational RCT evidence supporting vedolizumab use in UC, examining both strengths and limitations, and providing a comprehensive analysis of how artificial intelligence (AI), particularly machine learning (ML), can be leveraged to optimize vedolizumab treatment selection, predict outcomes, and personalize management.

**Methods:**

A systematic literature search was performed across PubMed, Scopus, and Web of Science databases. The review synthesized data from key Phase III trials (GEMINI 1, VARSITY), long-term extension safety studies, relevant meta-analyses summarizing efficacy and safety, and pertinent studies investigating the application of AI and ML techniques within inflammatory bowel disease management. The search included terms such as vedolizumab, UC, AI, and predictive modeling.

**Findings:**

Landmark trials confirmed vedolizumab’s superiority over placebo for inducing and maintaining remission, with week 52 clinical remission rates reaching 41.8% in the GEMINI 1 trial. Concurrently, emerging AI/ML models, integrating complex patient data, show considerable promise in predicting biologic response with high accuracy, with some models achieving an area under the curve (AUC) of 0.82 (95% CI 0.78–0.86). Neural networks have demonstrated an accuracy of approximately 79% in specific predictive contexts.

**Conclusions:**

The strategic integration of AI-driven predictive analytics with vedolizumab’s clinical and pharmacodynamic data represents a pivotal next step towards achieving true precision medicine in UC.

## 1. Introduction

The therapeutic landscape for ulcerative colitis (UC) has been significantly reshaped by biologics targeting specific inflammatory pathways [1]. Vedolizumab exemplifies this progress, functioning as a humanized monoclonal antibody that selectively inhibits the α4β7 integrin, thereby blocking lymphocyte trafficking specifically to the gut [1]. This targeted mechanism contrasts with the broader immunosuppression seen with earlier agents. Foundational trials, such as GEMINI 1, demonstrated substantial efficacy, reporting 52-week clinical remission rates reaching 41.8% during the maintenance phase [2]. Despite these results, clinical practice and real-world effectiveness studies reveal considerable interindividual variability in patient response, with many patients failing to achieve or sustain remission [3,4]. This discrepancy between trial efficacy and real-world effectiveness underscores two critical challenges that currently limit optimal UC management. The first relates to the inherent limitations of applying population-level averages derived from randomized controlled trials (RCTs) to predict treatment success for a specific individual patient, given the marked heterogeneity of UC. The second challenge is the current lack of validated and easily accessible biomarkers capable of reliably predicting, a priori, which patients are most likely to respond favorably to vedolizumab therapy [5].

Artificial intelligence (AI), and its subfield, machine learning (ML), offer powerful computational approaches to address these challenges. By processing vast and complex datasets that exceed human analytical capacity, AI and ML methods enable multidimensional data integration, synthesizing diverse inputs such as clinical history, patient-reported outcomes, endoscopic imaging scores, pharmacokinetic parameters including drug levels, and multi-omic data encompassing genomics, proteomics, and the microbiome to identify complex predictive patterns [6]. These approaches also support dynamic treatment optimization through the development of algorithms that may guide dose adjustments or suggest optimal treatment sequencing based on evolving patient data, with “dynamic dosing” referring to AI-guided dose modification informed by real-time patient information and predicted response. In addition, AI facilitates predictive model construction by enabling the development of sophisticated models capable of forecasting individual patient trajectories and estimating the likelihood of response to specific therapies such as vedolizumab [7,8].

This review will now proceed with a detailed analysis of the methodology used for the literature search, followed by a reappraisal of the evidence supporting vedolizumab’s efficacy and safety. It will then examine the applications of AI in personalizing vedolizumab therapy, including predictive modeling approaches and a proposed framework for clinical implementation. Finally, the review will address the challenges and limitations of AI, as well as the associated regulatory and ethical considerations, before offering conclusions regarding the future role of AI-guided vedolizumab therapy in UC.

## 2. Methodology

**Fig 1:**
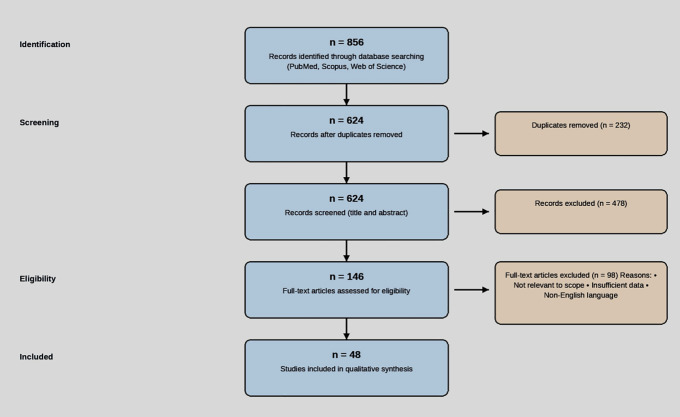
PRISMA flow diagram. PRISMA flow diagram illustrating the literature search and study selection process.

This review is based on a systematic search of the published literature. Major biomedical databases including PubMed, Scopus, and Web of Science were searched. Search terms included combinations of “vedolizumab,” “ulcerative colitis,” “inflammatory bowel disease,” “efficacy,” “safety,” “pharmacokinetics,” “artificial intelligence,” “machine learning,” “predictive modeling,” “biomarkers,” and specific trial names (e.g., “GEMINI,” “VARSITY”). The search included Phase III RCTs, long-term extension studies, meta-analyses, observational studies evaluating effectiveness and safety, and studies investigating AI/ML applications in IBD treatment prediction or optimization. Articles were selected based on relevance to the review’s objectives, focusing on vedolizumab in UC and the role of AI in personalizing therapy. The literature search and selection process is summarized in the PRISMA flow diagram below (Fig 1).

## 3. Vedolizumab efficacy and safety: Reappraising the evidence

A robust body of evidence supports vedolizumab’s role in UC, primarily derived from large-scale clinical trials and their extensions.

### 3.1 Induction and maintenance outcomes from key trials

The GEMINI 1 trial (*N* = 895) was a foundational Phase III, randomized, double-blind, placebo-controlled study that established vedolizumab’s efficacy for both induction and maintenance therapy. During the induction phase at week 6, a significantly higher proportion of patients receiving vedolizumab achieved a clinical response compared with placebo (47.1% versus 25.5%; *p* < 0.001) [2]. In the maintenance phase at week 52, among patients who responded at week 6, continued vedolizumab treatment resulted in significantly higher clinical remission rates compared with those who were switched to placebo (41.8% versus 15.9%; *p* < 0.001) [2].

The VARSITY trial (*N* = 769) was a Phase IIIb, double-blind, double-dummy, active-comparator study that provided the first head-to-head comparison between two biologic agents with different mechanisms of action in UC. At week 52, vedolizumab demonstrated superiority over the anti-TNF agent adalimumab in achieving endoscopic improvement (39.7% versus 27.7%; *p* = 0.006), a critical indicator of mucosal healing associated with improved long-term outcomes [9]. Clinical remission rates were statistically similar between the two agents [9]. Key efficacy outcomes from these pivotal trials are summarized in Table 1.

**Table 1:** Key efficacy outcomes from pivotal vedolizumab trials in ulcerative colitis.

Trial	Endpoint	Vedolizumab group (%)	Comparator group (%)	Comparator type	*P*-value
GEMINI 1	Week 6 Clinical Response (Induction)	47.1	25.5	Placebo	<0.001
	Week 52 Clinical Remission (Maintenance)^*^	41.8	15.9	Placebo	<0.001
VARSITY	Week 52 Endoscopic Improvement	39.7	27.7	Adalimumab	0.006
	Week 52 Clinical Remission	31.3	22.5	Adalimumab	Non-significant

*Maintenance analysis performed on patients who responded to vedolizumab induction at Week 6.

Despite their pivotal role, these trials have limitations that affect their direct translation to individual patient care. Notably, in the GEMINI 1 trial, nearly half of patients with prior anti-TNF exposure (48.9%) failed to respond to vedolizumab induction, highlighting the therapeutic challenge in this more refractory population [2]. In addition, deeper therapeutic targets such as histologic remission, defined as microscopic resolution of inflammation and potentially predictive of more durable outcomes, have largely been evaluated through post-hoc analyses rather than as primary trial endpoints [8]. Collectively, these limitations underscore the difficulty of predicting individual treatment response using baseline demographic or clinical variables alone and reinforce the need for more sophisticated, personalized predictive approaches.

### 3.2 Long-term safety profile

Understanding long-term safety (LTS) is crucial for chronic therapies such as vedolizumab. Data from the GEMINI LTS open-label extension studies have provided valuable insights into the LTS and tolerability of vedolizumab, with some observations spanning up to seven years, as referenced in related reviews [10]. Across extended periods of exposure, the overall safety profile remained generally consistent, with no major new safety signals emerging in long-term follow-up data synthesized in multiple reviews [11]. The incidence rate of serious infections was reported at approximately 5.5 events per 100 patient-years, a rate generally considered acceptable for this patient population [10]. Similarly, the malignancy rate appeared low, with earlier LTS analyses reporting approximately 0.6 events per 100 patient-years, comparable to background rates expected in inflammatory bowel disease cohorts [10].

Comparative safety data versus anti-TNF agents further highlight potential advantages of vedolizumab’s gut-selective mechanism. Meta-analyses and observational studies suggest a more favorable systemic safety profile for vedolizumab [11]. Systematic reviews have indicated that vedolizumab may be associated with a statistically significant lower risk of serious infections compared with systemically acting anti-TNF therapies, with hazard ratios of approximately 0.72 (95% CI 0.55–0.94) reported in some analyses [11]. This difference may have important implications for treatment selection, particularly in patients with an elevated baseline risk of infection.

## 4. AI-driven personalization of vedolizumab therapy

AI offers a transformative pathway to move beyond population-based evidence and tailor vedolizumab treatment by leveraging individual patient data complexity.

### 4.1 Predictive modeling approaches

The core of AI applications in this context lies in developing models that predict treatment outcomes. These models integrate diverse data types to capture individual patient heterogeneity. Key input variables used in successful models typically leverage a combination of clinical parameters, pharmacokinetic data, endoscopic features, and multi-omics biomarkers. Clinical parameters include baseline disease severity scores such as the Mayo score, disease extent, disease duration, prior treatment history, and longitudinal changes in symptoms, all of which have been shown to influence treatment response [3,4]. Pharmacokinetic data, particularly vedolizumab trough concentrations, provide additional insight into drug exposure, with early levels—such as week-6 concentrations exceeding 18 µg/mL—being associated with higher remission rates [12]. Endoscopic features derived from computer vision techniques allow for quantitative assessment of mucosal inflammation beyond standard scoring systems. Computer vision involves the automated analysis of endoscopic images and enables a more objective evaluation of inflammatory activity [7,13,14]. Multi-omics biomarkers, including genomics, transcriptomics, proteomics, and importantly the gut microbiome, may further enhance prediction by revealing underlying biological pathways that influence therapeutic response [15].

Leveraging these multidimensional inputs, early predictive models have demonstrated significant performance. Machine-learning algorithms such as Random Forests have achieved high discrimination, with reported areas under the curve of approximately 0.82 (95% CI 0.78–0.86) in predicting response to biologic therapies in inflammatory bowel disease [6]. Neural networks are also being explored for more dynamic tasks, including prediction of optimal vedolizumab dosing strategies, with early reports describing promising accuracies of around 79% in selected contexts, potentially enabling personalized dose adjustments [13].

## 5. Framework for clinical implementation

**Fig 2:**
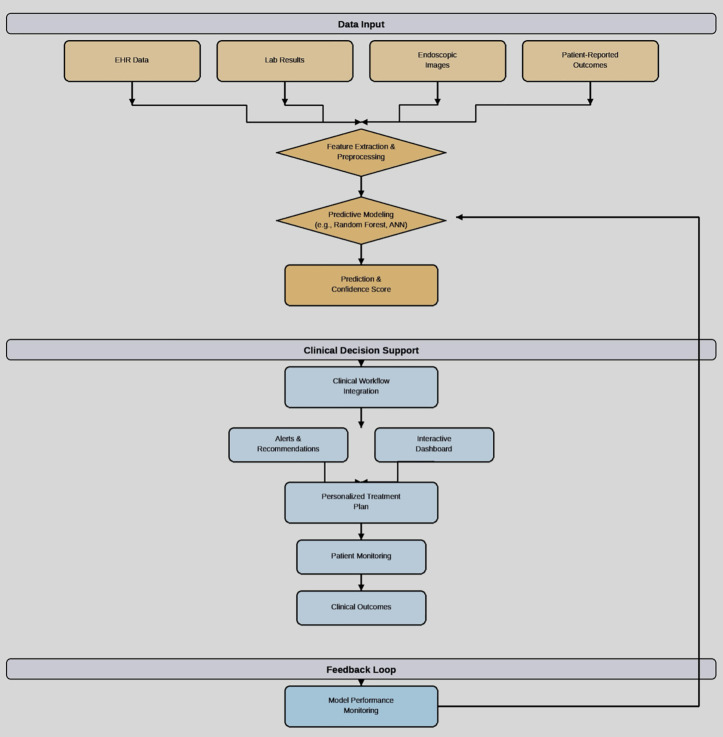
Clinical implementation framework. A proposed framework for the clinical implementation of AI-driven predictive models for personalizing vedolizumab therapy.

Successfully translating predictive AI models from research into routine clinical practice requires a robust and systematic implementation framework, as illustrated in Fig 2.

This framework highlights the key stages of data integration, AI-powered prediction, clinical decision support, and continuous model improvement. It emphasizes the importance of a feedback loop to ensure that the model adapts and improves over time as new data becomes available.

## 6. Challenges and limitations in AI implementation

While the potential of AI in personalizing vedolizumab therapy is immense, it is crucial to acknowledge the challenges and limitations that must be addressed before widespread adoption can occur. One key limitation relates to model generalizability, as AI models trained on specific populations may not perform reliably across diverse demographic groups, particularly underrepresented minorities in clinical trials, potentially leading to disparities in care if these issues are not adequately addressed. Overfitting represents another important concern, whereby models may perform well on training datasets but fail to maintain accuracy in real-world clinical applications, a risk that is particularly pronounced in complex models with many parameters. In addition, the performance of AI models is highly dependent on data quality, as incomplete, biased, or low-quality datasets can result in unreliable or misleading predictions. Finally, real-world performance drift must be considered, as the accuracy of AI systems may degrade over time with changes in clinical practice patterns and evolving patient populations, necessitating ongoing monitoring, validation, and periodic retraining to preserve model performance (Table 2).

**Table 2:** Overview of AI predictive modeling inputs and performance examples for vedolizumab response.

Data category	Key input variables (examples)	Performance metric example (illustrative)
Clinical	Baseline Mayo score, disease extent/duration, prior anti-TNF exposure	Component of multi-feature models
Pharmacokinetic	Week 6 trough concentration (>18 µg/mL associated with remission)	Predictive feature importance analysis
Endoscopic	Quantitative features from computer vision analysis (e.g., texture, vascular patterns)	Model accuracy improvement
Multi-Omics	Gut microbiome composition (e.g., specific fecal proteomic signature)	Identification of predictive biomarkers
Integrated Model Performance	Random Forest model integrating multiple data types	AUC = 0.82 (95% CI 0.78-0.86)^*^
	Neural Network for dose optimization	Accuracy ≈ 79%^*^

*AUC, Area Under the Curve (measure of model discrimination); Accuracy values reported in specific contexts/studies cited.

## 7. Regulatory and ethical considerations

The integration of AI into clinical decision-making raises important regulatory and ethical questions that must be carefully considered. One key requirement is compliance with U.S. Food and Drug Administration guidance on Software as a Medical Device (SaMD), which outlines expectations for algorithmic transparency and validation; developers of AI models intended for clinical use must adhere to these regulatory standards. In parallel, algorithmic transparency is essential for clinical adoption, as clinicians must be able to understand how AI models arrive at their predictions. This necessitates the use of explainable AI techniques that provide interpretable insights into the model’s decision-making process. Bias mitigation represents another critical consideration, as AI systems can perpetuate or amplify existing biases present in healthcare datasets. Consequently, robust strategies for identifying, monitoring, and mitigating algorithmic bias are required to promote equitable care delivery. Finally, the deployment of AI in healthcare demands well-defined data governance protocols to ensure patient privacy, data security, and ethical use of clinical information. Such frameworks should explicitly address informed consent for the use of patient data in AI model development, validation, and ongoing refinement.

## 8. Future directions

Realizing the full potential of AI-guided vedolizumab therapy necessitates continued research, development, and careful consideration of practical challenges across several key domains. Future research should prioritize prospective clinical trials designed to validate both the clinical utility and cost-effectiveness of AI-guided therapeutic strategies. In parallel, further exploration of multi-omics data is required to identify novel biomarkers that can enable patient stratification and improve the prediction of treatment response. In addition, ongoing dialogue and focused research are essential to address the ethical challenges associated with deploying AI in clinical decision-making, including issues related to accountability, liability, and preservation of patient autonomy.

## 9. Conclusion

Vedolizumab has significantly improved outcomes for many patients with moderate-to-severe UC, offering a gut-selective mechanism with a favorable safety profile. However, primary non-response remains a substantial clinical challenge, driving the need for more personalized approaches. This review highlights the considerable potential of AI and ML to address this need. By integrating complex, multidimensional patient data—spanning clinical, pharmacokinetic, endoscopic, and multi-omic domains—AI models are emerging as powerful tools capable of predicting individual treatment response with increasing accuracy. The path forward involves rigorous prospective validation of these predictive models in diverse patient populations, and their careful integration into clinical practice through user-friendly decision support systems. Successfully navigating the regulatory, ethical, and economic hurdles will be essential. Ultimately, the synergy between advanced data analytics and targeted biologic therapy like vedolizumab holds the promise of ushering in a new era of precision medicine for UC, enabling clinicians to optimize treatment selection, improve patient outcomes, and enhance healthcare value.
